# Determination of the Botanical Origin and Physicochemical Properties of a Propolis Sample Through an Integrated Methodology

**DOI:** 10.3390/antiox13111412

**Published:** 2024-11-18

**Authors:** Jose Juan Alcivar-Saldaña, Marco Aurelio Rodriguez-Monroy, Arturo Aguirre-Gómez, Liborio Carrillo-Miranda, Benjamin Velasco-Bejarano, Maria Margarita Canales-Martinez

**Affiliations:** 1 Laboratorio de Farmacognosia, Unidad de Biotecnología y Prototipos (UBIPRO), Facultad de Estudios Superiores Iztacala, Universidad Nacional Autónoma de México (UNAM), Avenida de los Barrios Número 1, Colonia Los Reyes Iztacala, Tlalnepantla de Baz 54090, CP, Mexico; bio.alcivar@gmail.com; 2 Posgrado en Ciencias Biológicas, Universidad Nacional Autónoma de México, Unidad de Posgrado, Edificio D, 1° piso, Circuito de Posgrado, Ciudad Universitaria, Coyoacán 04510, CP, Mexico; 3 Laboratorio de Investigación Biomédica en Productos Naturales, Carrera de Medicina, Facultad de Estudios Superiores Iztacala, Universidad Nacional Autónoma de México (UNAM), Avenida de los Barrios Número 1, Colonia Los Reyes Iztacala, Tlalnepantla de Baz 54090, CP, Mexico; dr.marcorodriguezmonroy@gmail.com; 4 Laboratorio de Análisis e Investigación en Química Agrícola y Ambiental, L-15 UIM, Departamento de Química Campus 4 Facultad de Estudios Superiore Cuautitlán, Universidad Nacional Autónoma de México, Carretera Cuautitlán-Teoloyucan km. 2.5, Col. San Sebastián Xhala, Cuautitlán Izcalli 54714, CP, Mexico; aag@unam.mx; 5 Módulo de Apicultura, Centro de Enseñanza Agropecuaria (CEA), Facultad de Estudios Superiores Cuautitlan, Universidad Nacional Autónoma de México, Carretera Cuautitlán-Teoloyucan km. 2.5, Col. San Sebastián Xhala, Cuautitlán Izcalli 54714, CP, Mexico; liboriocami@gmail.com; 6 Laboratorio de Química Verde Medicinal, Departamento de Ciencias Químicas, Facultad de Estudios Superiores Cuautitlan, Universidad Nacional Autónoma de México, Av. Primero de Mayo s/n, Sta. María Guadalupe las Torres, Cuautitlán Izcalli 54740, CP, Mexico; qfbbenjamin.velascob@cuautitlan.unam.mx

**Keywords:** *Apis mellifera*, plant sources, pollen, phenols, flavonoids

## Abstract

The growing interest in products of natural origin has led to the implementation of products such as propolis because they possess biological properties that are useful in the treatment of various ailments, so it is relevant to know the botanical origin of the physicochemical compounds that provide propolis with its biological properties. To identify the floral sources that provide resources to bees for the manufacture of propolis, several methodologies have been implemented, such as palynological analysis, which, through pollen content, has made it possible to identify the plant species that provide resources to bees. On the other hand, analysis of the physicochemical components of propolis has revealed that phenols and flavonoids are mainly responsible for the biological activity of propolis. Evaluation of the antibacterial and antifungal potential of propolis revealed the inhibitory potential of both Gram (+) and Gram (−) bacteria, as well as *Candida albicans*. However, all these investigations have been carried out individually and not always with the same sample. Therefore, the objective of this research was to design a methodology that allows the use of a single sample of propolis and uses sufficient resources for different research areas to evaluate most of the physical and chemical properties of propolis, as well as its botanical origin. With the implemented methodology, it was possible to obtain sufficient resources that provided results for each of the research areas, taking advantage of the propolis sample.

## 1. Introduction

The growing interest in products of natural origin for food, therapeutic or medicinal purposes has led to the use of those generated by honeybees and other native bee species (*Melipona quadrifasciata*, *Melipona compressipes*, *Tetragonisca angustula* and *Nannotrigona* sp.) [1,2,3,4,5]. Among these products, propolis stands out; it is a dark-colored resin manufactured by bees from resins and balsams produced by various plant species and processed by *Apis mellifera* L., which functions as a thermal insulator and antiseptic for this species [3,6]. After the process of collection, transport and storage, in the hive, the bees add enzymes and glandular secretions from the hypopharynx, as well as waxes from the wax glands present in the sternites of the abdomen, forming a final product that differs in its chemical composition from one hive to another and from one species to another [3,4].

Propolis and geopropoleum are recognized for their antifungal, antibacterial, anticancer, antiprotozoal, anesthetic, tissue regeneration, antiviral and natural antioxidant properties [3,4]. However, both the physical and chemical properties of propolis are closely related to the vegetation that provides honeybee resources, as well as the climatic conditions in which this product is manufactured [2,7]. This vegetation provides phytochemical compounds such as phenolic compounds, esters, phenolic acids and flavonoids such as apigenin, pinocembrin, chrysin, hesperidin, quercetin, kaempferol and galangin, which, together with the enzymatic contributions of honeybees added during the manufacturing process, enrich propolis [7,8].

Several studies have aimed to identify the vegetation and source of resources for the manufacture of this product, such as observing the foraging activity of honeybees and performing palynological analysis and genetic determination of the pollen content of propolis [2,9,10,11,12]. Palynological analysis is the most widely used method suitable for the melizopalynological analysis of honey, which has a relatively liquid consistency and is easy to dissolve in water or other solvents such as ethanol, facilitating the extraction of pollen, but in the case of propolis, which has a gummy and resinous consistency, the implementation of this method is somewhat complicated for the extraction of pollen. Therefore, various methodologies have been designed with the aim of extracting the greatest amount of pollen from propolis [13].

One of the methodologies implemented for pollen extraction is sonication, which, by means of ultravibrations, disintegrates the components of propolis [14]. Another regularly implemented method is total cleaning by Soxhlet, in which the propolis sample is leached with ether and then with 96° ethanol [14]. At the end of both techniques, the residues are treated via the acetolysis technique [15], which eliminates any organic residue from the sample, leaving only pollen structures, which are resistant because they are composed of sporopolenin, a protein that resists acid treatment [16]. However, these methods have several drawbacks, such as damage to the pollen structure caused by sonication, making the taxonomic identification of pollen difficult. In turn, in studies that implement the acetolysis technique [15], which is correct for performing more accurate determinations of pollen types, its drawback is the acidic nature of the process, which eliminates other biological structures of interest when it comes to gaining knowledge about the constitution and quality of propolis, such as spores, fungi, bacteria and other structures that provide information of interest [17]. For the Soxleth method, the drawback lies in the amount of sample to be analyzed, since with small samples (1 g), the pollen content obtained is low, with a minimum representation of the total diversity of pollen types present in the sample [14].

Therefore, it is necessary to implement extraction methods that focus on the physicochemical characteristics of propolis, which manage to disintegrate the conglomerate of compounds of which propolis is constituted and allow not only the extraction of pollen but also the acquisition of useful resources in the analysis of other areas of study. Some methods that could be of interest have been designed and implemented in biochemistry to extract compounds from samples of different herbaceous plants, roots or bark, in which the polarity of the phytochemical components is considered to implement solvents that extract these compounds [18]. Therefore, considering the constitution of propolis is composed mainly of phenols, flavonoids and waxes, several investigations have implemented the use of solvents such as water, ethanol, methanol or hexane to obtain a soft extract of propolis, which is implemented in analyses such as phytochemical characterization by high-performance liquid chromatography (HPLC), in antibacterial and antifungal evaluation or as an active compound in the elaboration of products for therapeutic purposes [4,9,17,18]. However, although each area provides results of interest for gaining knowledge of the constitution and activity of propolis, there are few investigations that work on a propolis sample in an integral way. Therefore, the present research proposes a methodology based on the physicochemical characteristics of propolis, which allows for the analysis of resources for phytochemical evaluation and characterization, antibacterial and antifungal evaluation, and sufficient material for palynological analysis, which allows for an integral analysis of the relevant sample, resulting in the physicochemical characterization and botanical origin of the studied propolis.

## 2. Materials and Methods

### 2.1. Study Area

The propolis sample was provided by the Universidad Nacional Autónoma de México (UNAM) at the Facultad de Estudios Superiores Cuautitlan (FES-C), located in the Estado of Mexico, municipality of Cuautitlan Izcalli, Mexico (19°41′; north, 99°11′ west, 2260 m a.s.l.), within the highland beekeeping region [19].

### 2.2. Sample Collection Method

**Figure 1:**
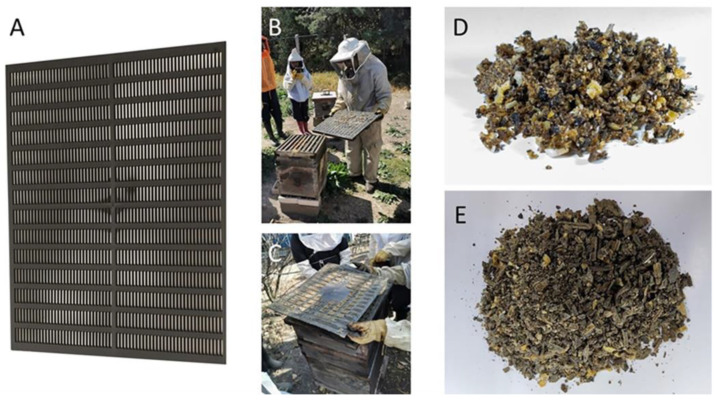
Propolis collection method at FES-C. (**A**) PC; (**B**) location of PC; (**C**) removal of PC; (**D**) sample obtained without PC with too many impurities; (**E**) sample obtained with PC with few impurities.

**Figure 2:**
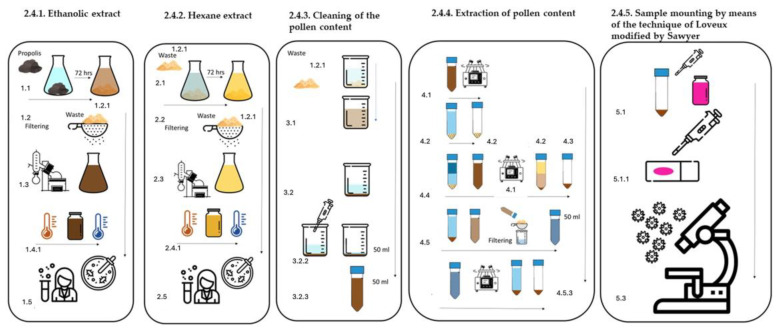
Methodology for obtaining EEP and EHP, as well as pollen content.

The sample was obtained by means of a propolis collector (PC) (Brand propolistrap, MI, USA) (Figure 1); such a collector is a high-density polystyrene grid of black color that is placed over the brood chamber or supers according to the case, which, after a certain time and at the beekeeper’s consideration or the filling of the PC, is removed. Once the PC is out of the apiary, it is frozen at −20 °C; for its extraction, the propolis frozen in the PC is removed via cryofracture, resulting in what is called propolis in shreds (PS) (Figure 1). Due to the implementation of the PC, the collected propolis presented less than 10% impurities; thus, 100 g of this material was taken for the extraction process according to the proposed work scheme (Figure 2) [20].

### 2.3. Pollen Extraction via the Soxhlet Method

The propolis sample was processed via Soxhlet extraction, which removes both resins and waxes from a gram of propolis by leaching it with 96% ethanol to remove resins and petroleum ether to remove waxes and fats [21,22].

### 2.4. Extraction Methods with Solvents of Different Polarities

#### 2.4.1. Ethanolic Extract of Propolis (EEP), (Figure 2) [18]

1.1 One hundred grams of PS was placed in a flask with 70% ethanol at a 1:5 ratio and covered at room temperature in the dark for 72 h.
1.2. After 72 h, the contents of the flask were filtered through Whatman filter paper to obtain an ethanolic extract.
  1.2.1. The residue obtained after the filtration process was preserved.
1.3. The ethanolic extract obtained was distilled via a rotary evaporator at 40 °C to ensure that the extract was dry.
1.4. The extract obtained from the distillation mixture was brought to total dryness at room temperature, after which it was spread in glass containers and named ethanolic extract of propolis (EEP).
  1.4.1. Once the totality of the solvent was evaporated, it was stored in closed glass jars and kept frozen at −20 °C.
1.5. The extract obtained was used in individual portions for physical and chemical analysis, according to the quantities indicated in each technique.

#### 2.4.2. Hexane Extract of Propolis (EHP) (Figure 2) [18]

2.1. The residues obtained from the filtration process in Step 1.2.1 were placed in a flask with hexane at a 5:1 ratio and covered at room temperature in the dark for 72 h.
2.2. After 72 h and inside an extraction hood, the contents of the flask were filtered through Whatman filter paper to obtain a hexanic extract.
  2.2.1. The residue obtained after the filtration process was preserved.
2.3. The obtained hexanic extract was distilled Via a rotary evaporator at 26 °C to ensure that the extract was dry and named hexane extract of propolis (EHP).
2.4. The hexanic extract obtained from the distillation was brought to total dryness in an extraction hood at room temperature, and the extract was extended in glass containers.
  2.4.1. Once the totality of the solvent was evaporated, it was stored in closed glass jars and kept frozen at −20 °C.
2.5 The obtained hexanic extract was used in individual portions for physical and chemical analysis according to the quantities indicated in each technique.

#### 2.4.3. Cleaning of the Pollen Content (Figure 2)

3.1 The residues obtained from the filtering process described in Step 2.2.1 were disintegrated in 70% ethanol and shaken vigorously until the mixture was homogenized.
3.2. The obtained mixture was filtered through sterile gauze, which retained persistent impurities, as well as resins not dissolved in the medium.
  3.2.1. The previous process was carried out many times until a fine residue that was left to precipitate was obtained.
  3.2.2. Once the precipitate formed, the solvent was withdrawn until a volume of 50 mL was obtained.
  3.2.3. The obtained volume was deposited in a 50 mL centrifuge tube.

#### 2.4.4. Extraction of Pollen Content (Figure 2)

4.1. The mixture obtained at point 3.2.3 was centrifuged at 2500 rpm for 10 min.
4.2. At the end of the centrifugation process, the supernatant was removed, and the pellet, which was a wax matrix with pollen, was retained (Figure 2).
4.3. Recovery of the pollen content of the wax–pollen matrix.
4.4. The pellet (wax-pollen matrix) inside the 50 mL centrifuge tube was disintegrated in 25 mL of 96% ethanol. Once the pellet was dissolved, 25 mL of hexane was added to the tube, and it was shaken gently until both solvents were incorporated.
  4.4.1. Once the sample was homogenized, it was centrifuged at 2500 rpm for 10 min.
  4.4.2. After this time, a double phase is formed where the elements are separated in the following order from bottom to top: pollen tablet and some impurities, ethanol, hexane with dissolved waxes, and persistent resins (Figure 2).
  4.4.3. To finalize the process of obtaining pollen, the liquid phase was removed, and the pellet was preserved.
4.5. The obtained pellet disintegrated in 70% ethanol, with the purpose of removing the impurities that the pollen grains could have accumulated on the ornamentation of the exine of each species.
  4.5.1. The disintegrated pellet was filtered with gauze, the filtrate obtained was deposited in a centrifuge tube, and if necessary, it was gauged to 50 mL with 70% ethanol.
  4.5.2. Later, the sample was centrifuged at 2500 rpm for 10 min.
  4.5.3. After centrifugation, the pellet was recovered, and the supernatant was removed.

#### 2.4.5. Sample Mounting via the Technique of Loveux [21] Modified by Sawyer [22]

5.1 With the aid of a micropipette, 100 µL of glycerogelatin with basic fuchsin was taken and added to the pellet.
  5.1.1. With the aid of a micropipette, the gelatin is retracted with pieces of the pellet many times until the mixture is homogenized.
5.2. Place the homogenized mixture on an object holder with the help of an object cover to distend the mixture.
  5.2.1. Once the sample is mounted, it is sealed with transparent varnish.
5.3. The obtained slides were reserved for palynological analysis.

The slides were examined under a Nikon Labophot-2 optical microscope at 40× and 100× [21,22]. To determine the pollen grains, dichotomous keys and specialized studies in the literature were used. With the determination of pollen content, the percentage of pollen types was determined via the method proposed by Loveux [21] and modified by Sayer [22]; four equidistant transects in the width of the preparations were marked, which served as guides where 500 pollen grains were counted per transect, in which the occurrence of each pollen type was recorded, to establish the pollen percentage.

### 2.5. Physicochemical Analysis

#### 2.5.1. Waxes

The concentration of wax was determined by the difference in weight between the weight of the propolis in the shag and the weight of the EHP extract, with the percentage obtained not being greater than 25% [23,24]. The yield was calculated according to the following equation [24]:

$$R\left(\%\right)=\frac{P}{m}(100)$$

*R* = yield in percent

*P* = weight of the extract in (g)

*m* = Initial weight of the sample (g)

#### 2.5.2. Total Phenols

The concentration of total phenols was determined through the modified Singleton method [25] by spectrophotometry based on an oxidation–reduction colorimetric reaction using Folin–Ciocalteu’s reagent as the oxidizing agent and measuring absorbance at 760 nm [26,27].

##### Calibration Curve

A 0.2 mg/mL gallic acid standard solution was used for the calibration curve. From this solution, serial aliquots of 0.00625, 0.0125, 0.05, 0.1, and 0.2 mg/mL of gallic acid were made, with roughly 1 mL with distilled water [26,27].

##### Preparation of EEP

An amount of 250 mg of EEP dissolved in 2 mL of distilled water was prepared by taking an aliquot of 250 µL and adding 750 µL of distilled water to obtain a concentration of 1 mL [26,27].

##### Analysis

To each concentration of gallic acid and the propolis (EEP), 7 mL of distilled water and 500 mL of the Folin–Ciocalteu reagent were added. After 5 min, 1.5 mL of Na_2_CO_3_ solution (200 g/L) was added, allowing it to stand for 2 h at room temperature and then taking the absorbance reading at 760 nm [26,27].

#### 2.5.3. Total Flavonoids

Dowd’s method determined the total flavonoid content of propolis [27,28].

##### Calibration Curve

An amount of 3 mg of quercetin dissolved in 3 mL of HPLC grade methanol were prepared, then aliquots were taken to form a standard curve with concentrations ranging from 1 to 100 ppm.

##### Preparation of EEP

A total of 100 mg of EEP was weighed and dissolved in 3 mL of methanol to form the stock. Subsequently, 1 mL of the stock was taken and 1 mL of 2% AlCl_3_ was added. The blank sample consisted of 1 mL of the stock without AlCl_3_ [27].

##### Analysis

The standard curve and the sample were allowed to stand at room temperature for 10 min, and then the absorbance was read at 450 nm [27].

#### 2.5.4. Antioxidant Capacity

To evaluate this parameter and calculate the antioxidant capacity of propolis at 50% (IC_50_), the reduction of the 2,2-diphenyl-1-1picrihydrazyl radical (DPPH) was carried out by means of the technique proposed by [29,30,31]. For the preparation of the sample, 100 mg of EEP dissolved in 5 mL of methanol (HPLC) was used, while the blank consisted of 200 μL of methanol. The absorbance of each sample was subsequently measured at 540 nm on an ELISA plate reader (Multiskan-Ex Thermo Scientific, Waltham, MA, USA). The results are reported as the percent reduction via the following equation:

$$\%\;Reduction=\frac{C-E}{C}\left(100\right)$$

where

C = absorbance of DPPH;

E = Absorbance of the experiment (DPPH + propolis mixture).

### 2.6. Chromatography Analysis of the Propolis

#### 2.6.1. Gas Chromatography–Mass Spectrometry Ethanolic Extract

The ethanolic extract that was obtained from the maceration of the propolis in Section 2.4.1 was analyzed by gas chromatography–mass spectrometry using a Model 6850 chromatograph (Agilent Technologies, Santa Clara, CA, USA) coupled to a Model 5975C mass spectrometer (Agilent Technologies) with an HP-5MS column (30 m × 0.25 mm, 0.25 µm Agilent Technologies). An amount of 1 µL of the sample was analyzed from a 3 mg/mL solution injected in split mode at an initial temperature of 70 °C for 2 min followed by a heating ramp of 15 °C min^−1^ at 290 °C for 6 min, using helium as a carrier gas for a total time of 31 min. The mass range detected was 35–600 m/z, ionizing by electron impact at 70 eV at an ionization temperature of 230 °C. The compounds were identified by comparison with the NIST library database version 8.0 (National Institute of Standards and Technology Gaithersburg, MD, USA).

#### 2.6.2. High-Performance Liquid Chromatography–Diode Array (HPLC-DAD) and HPLC-ESI-TOF-MS

The ethanolic extract of the studied propolis was also analyzed by means of the HPLC-DAD system (Hewlett Packard, Agilent Technologies 1100 Wilmington, DE, USA) equipped with a diode array detector (DAD) 1100 operated by Chen Station A0903. A total of 20 µL of the propolis sample was analyzed from a 3 mg/500 μL injected solution. The mobile phase consisted of methanol–acetonitrile–H_3_PO_4_–H_2_O (25:25:0.1:50) under isocratic conditions for 35 min, a procedure that provided better resolution when injecting the flavonoid standards from the database, using an Allsphere ODS-1 column (250 mm × 4.6 mm, with a particle size of 5 µm) at a pressure of 269 bar and a temperature between 22 and 23 °C. In comparison, the flow rate was 1 mL/min. A 280 nm wavelength diode array detector (DAD) with a full 200–400 nm scan was used. Detected compounds were identified by comparing the retention time and their absorption maxima (λmax) under ultraviolet light of the standards. The standards that made up the database were luteolin, genistein, caffeine, apigenin, myricetin, chrysin, acacetin, kaempferol, catechin, pinocembrin, baicalein, naringenin, naringin, catechol, and quercetin of the Sigma-Aldrich brand (St. Louis, MO, USA). HPLC-ESI-TOF-MS was performed with an Agilent 1200 Infinity coupled to an Agilent 6230 TOF mass spectrometer with an Agilent dual ESI source (ESI SG1 4289023) and the software Mass Hunter Workstation, version B.05.01, Build 5.01.5125.3, in negative ionization mode. The capillary voltage was 4000 V; the dry gas temperature was 250 °C; nitrogen was used at a flow rate of 6 l min^−1^; the nebulizer pressure was 60 psi; the MS range was 50–1300 *m*/*z*; and the Ms acquisition rate was one spectra/s. Chromatographic separation was performed using an HPLC system (Infinity Series 1200, Agilent Technologies, Waldbronn, Germany) equipped with a Kinetx 2.6 U, C18 100 Å (150 × 2.1 mm) column (Phenomenex, Torrance, CA, USA).

### 2.7. Evaluation of Antimicrobial Activity

The antimicrobial activity of EEP against *Gram* (+) and *Gram* (−) bacterial species as well as against yeast fungi was evaluated. This effect was evaluated via the Kirby–Baüer method of the CLSI [32,33], in which agar plates were inoculated with known concentrations of the microorganisms *Staphylococcus aureus* ATCC 25923, *Escherichia coli* ATCC 123 and *Candida albicans* ATCC 10231.

The suspension with microorganisms was prepared as follows: a roast of the colony to be evaluated was taken and immersed in Müeller–Hinton (MH) broth for bacteria and Sabouraud broth for yeasts. The tubes containing the inoculated broth were incubated at 37 °C for 24 hr. The surface of each agar plate was subsequently inoculated (bacteria 1–1.5 × 10^8^ CFU/mL; yeast (1–1.5 × 10^6^ CFU/mL)), seeded via the streaking method in three directions, and the plate was rotated at 60° angles at the end of each streak. Once the inoculum was dry, 6 mm diameter sensidiscs impregnated with 4 mg (400 mg/mL) of EEP per sensidisc were placed; as a positive control, sensidiscs with 25 μg of chloramphenicol for bacteria and with 25 μg of Nystatin for yeast fungi were placed; as a negative control, sensidiscs with 10 μL of 80% ethanol were placed.

## 3. Results

### 3.1. Methodology

#### 3.1.1. Pollen Extraction via Soxhlet

**Figure 3:**
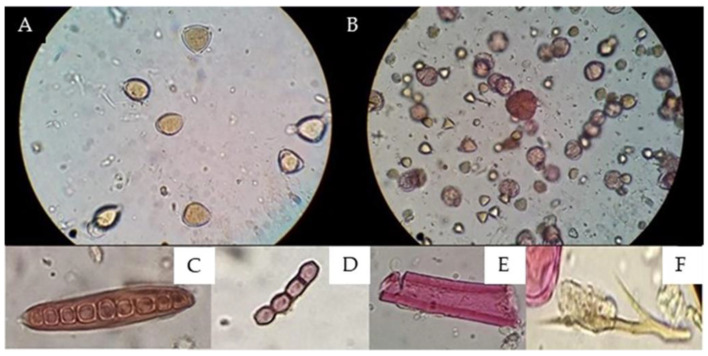
Comparisons of the results obtained via microscopic observations between the (**A**) Soxhlet method [21,22] and (**B**) proposed methodology. (**A**) Field 400X magnification without observing coloration changes due to the action of fuchsin on pollen grains. (**B**) Field 400X magnification coloration changes due to the action of fuchsin, (**C**) fungal spores, (**D**) spores, (**E**) plant structure, and (**F**) animal str.

The Soxhlet method resulted in the efficient extraction of pollen grains under good conditions for observation and the absence of waxes and resin, and no extracts were obtained for another analysis (Figure 3).

#### 3.1.2. Extraction Methods with Solvents of Different Polarities

The methodology proposed in Figure 2 allowed the pollen contained in the propolis to be obtained via extraction of its constituents, such as phenols, flavonoids, and waxes, via solvents according to the polarity of these components. With this methodology, two ethanol (EEP) and hexane (EHP) extracts were also obtained, which from 100 g of propolis sample in shaggy propolis yielded 25.20% EEP, with which physicochemical and biological analyses were carried out. For the EHP yield, 11.11% was obtained as the wax concentration in the 100 g propolis sample in the shaggy propolis. The extracts to be implemented in other research protocols, such as physicochemical evaluation and antibacterial and antifungal activity, were preserved and frozen at −20 °C.

The implementation of gauze resulted in the extraction of pollen content, since the adsorbent capacity of the gauze retained both resins and waxes and even persistent impurities that prevented adequate observation, as shown in Figure 3; in turn, the wide light of the gauze gave way to other different particles, such as pollen, fungi, and bacteria.

**Figure 4:**
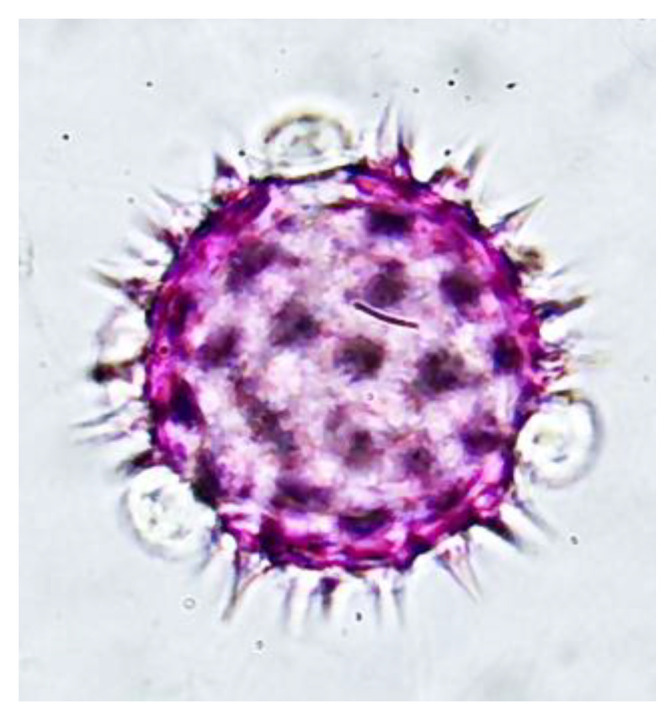
Pollen grain of the family Asteraceae in the genus *Bidens* obtained via the proposed extraction method 1000X magnification.

The double solvent phase process, 96° ethanol and hexane, resulted in the disintegration of the phenolic and serum components of the propolis residues, separating the pollen content from the wax–pollen matrix, which, together with washing with 70% ethanol, decreased the concentration and cleaning of the pollen content, which was reflected in the higher optical resolution of the pollen grains observed in Figure 4.

Staining with basic fuchsin incorporated into glycerogelatin (mounting medium) allows palynological analysis to differentiate between pollen grains and other structures, such as fungi and bacteria, because their composition is not stained by basic fuchsin (Figure 3).

### 3.2. Palynological Analysis

#### 3.2.1. Soxhlet Method

Mounting of the residues with pollen was carried out by means of glycerogelatin with basic fuchsin to obtain semipermanent samples, which were used for optical analysis, resulting in a total of 13 families, 17 genera, and 13 species (Table 1), not finding a main pollen type according to the classification proposed by Loveux [21] and modified by Sayer [22], with the pollen of *Brassica rapa* L. (IZTA-3814) being the most represented species, with 40%, followed by *Melaleuca citrina* (Curtis) Dum. (IZTA-3814), with a value of 10.56% (Table 2).

#### 3.2.2. Extraction Methods with Solvents of Different Polarities

By means of this method, a total of 22 families, 32 genera, and 21 species were determined (Table 3), with the *Brassica* genus being the predominant pollen type with 63.8%, followed by the Mirtaceae family with 5%. The percentages of the other taxonomic groups were less than 5% (Table 4).

### 3.3. Physicochemical Analysis

The results for total phenols showed a concentration of 62.79 mg GAE-g^−1^, which is expressed as a percentage equivalent to 6.27%, whereas the result for total flavonoids the concentration was 6.72 µg Qe-g^−1^, which is 0.61% of the concentration of this type of compound. For antioxidant activity, an IC_50_ of 486.43 µg/mL was determined.

### 3.4. Chromatographyl Analysis of the Propolis

#### 3.4.1. High-Performance Liquid Chromatography–Diode Array (HPLC-DAD) and HPLC-ESI-TOF-MS)

**Figure 5:**
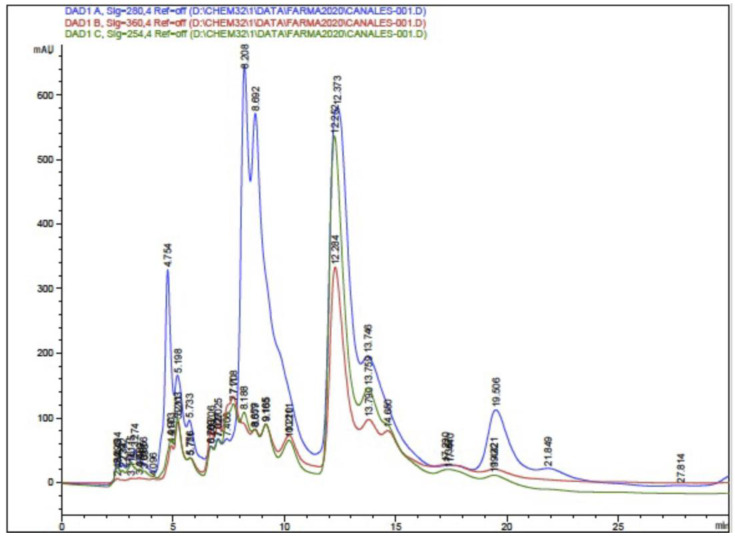
Chromatogram of ethanolic extract of propolis HPLC-DAD.

**Figure 6:**
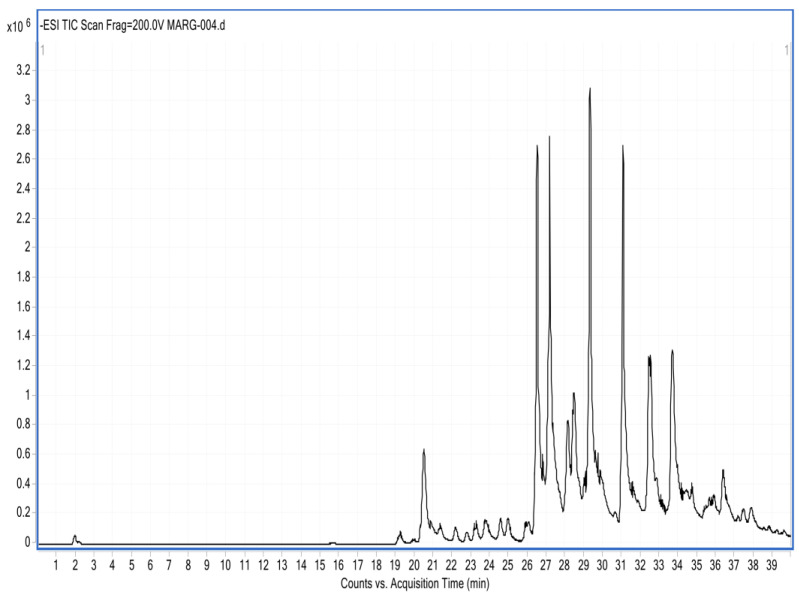
Chromatogram of the ethanolic extract of propolis HPLC-MS.

The HPLC-DAD analysis showed 17 compounds, 5 of which coincided with the database, compounds that are in the group of phenols and flavonoids. In the HPLC-MS analysis, 17 compounds were also determined, with 4 matches with the database, as shown in Table 5 and Figure 5 and Figure 6. Mass spectra of the compounds are available in Figure S1 of the Supplementary Material.

#### 3.4.2. Gas Chromatography–Mass Spectrometry (GC-MS)

**Figure 7:**
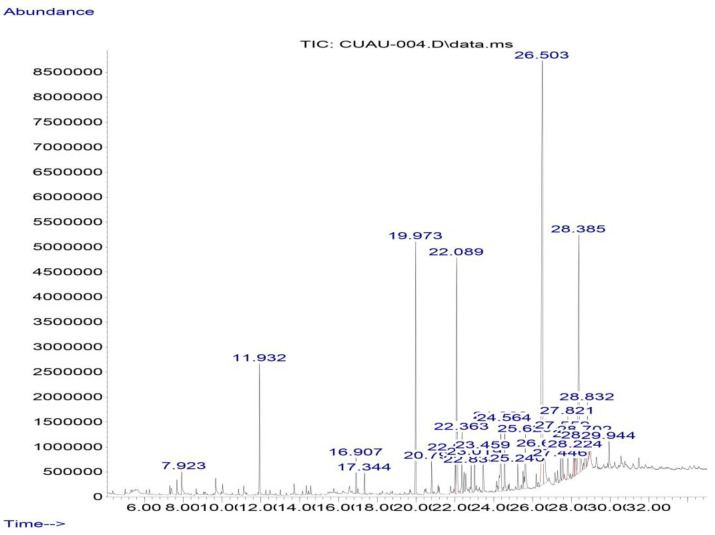
Chromatogram of the ethanolic extract of propolis GC-MS. Pinocembrin (RT = 26.5 min) and chrysin (RT = 28.39 min)are the most abundant flavonoids.

In the gas chromatography–mass spectrometry analysis of the ethanolic propolis extract sample, 16 compounds were identified, including pinocembrin, as shown in Table 6 and Figure 7.

#### 3.4.3. Biological Analysis

The antibacterial analyses revealed an inhibitory effect on *S. aureus*, with an inhibition halo of 8.71 mm; however, no inhibitory activity was observed for *E. coli*. In the evaluation of antifungal activity, no inhibitory activity was observed against *C. albicans*.

## 4. Discussion

The methodology suggested in this research allowed the pollen content with which qualitative and quantitative palynological analyses were carried out to be obtained from the propolis samples because the pollen grains were obtained under optimal physical conditions for determination. In turn, the differential extraction of the propolis components allowed the extraction of extracts with which biological and physicochemical tests were performed [18,22,26,34,35,36]. Therefore, this tool is proposed as a practical method for studies aimed at the analysis and palynological characterization of the physicochemical and biological properties of propolis [18,36].

The pollen sample obtained was abundant, representative, and in good condition for palynological analysis, which allowed more options for comparison and the precise taxonomic determination of each of the pollen species present in the sample [37,38]. With taxonomic knowledge of the pollen content, it was possible to infer the botanical origin of propolis, which is useful in the study, classification, and commercialization of propolis, as well as a better knowledge of its biomedical properties [37].

The washing process with solvents of different polarities described in point 2.4 of the proposed methodology allowed a greater number of pollen grains to be released from the conglomerate of waxes, resins, and balsams that constitute the propolis. In turn, the pollen grains were observed to be more clean and to have fewer impurities than with other methods, allowing high-resolution images in which the pollen structures useful for determination with dichotomous keys are detailed to be obtained, so that the results obtained both in concentration and resolution differ from the results obtained by the Soxhlet method proposed by Loveux [21] and modified by Sayer [22], while presenting the advantage of obtaining extracts useful for physicochemical and biological analysis [18,22].

In the design of the processes of this methodology, the acetolysis technique was not included, which, although it has the purpose of concentrating and specifying the determinations of pollen content by means of an acid train, also eliminates the totality of the biologicals present in the sample, eliminating the possibility of identifying organisms and structures whose determination and quantification would be a parameter that would promote the quality of the propolis. Thus, with the mounting process described in this method, it is possible to observe fungal spores, yeasts, and plant and animal structures under an optical microscope [15,33]. However, in future studies and to improve the determination of pollen types at specific taxonomic levels, such as genus and species, the pollen material obtained at point 4.5.3 of the methodology should be reserved and subjected to acetolysis [15].

The EEP and EHP obtained by using solvents concentrate the main constituents of propolis, such as phenols, flavonoids and waxes [6,18]. Therefore, such extracts are useful in various research protocols, especially in the determination of the physical and chemical composition of propolis. In turn, the same extracts used in biological activity protocols generate results for antibacterial action [39].

The analysis of EHP is a quality parameter of propolis, since the concentration of wax in propolis does not present components that can promote any biological property; thus, it can be taken as a residue, included by the bees in the manufacturing of this product or included by the beekeeper in processes not suitable for obtaining propolis, because its inclusion reduces the volume of resins and balsams that are sources of phenolic compounds, which promote the biological properties of propolis. Therefore, the propolis sample studied with a wax concentration of 11.11% can be considered good-quality propolis since it is less than 25%, which is the maximum limit for considering the sample to be of good quality [26].

The pollen content allowed for the palynological analysis of the sample, revealing that the main pollen type that constitutes the propolis of the FES-C corresponds to the pollen of the *Brassica* genus, which is the main pollen type, followed by *M. citrina* and *E. globulus*, suggesting that the constitution of the propolis sample of the FES-C presents not only resins belonging to these two species but also a high content of balsams from species of the *Brassica* genus, such as *B. rapa,* which would explain the green color of this propolis, as suggested by Londoño et al. [40], who specify that the coloration of propolis depends largely on the propolis flora so that propolis that includes balsams in their constitution usually presents traces or green tones instead of a brown color, which is more common in propolis. These results contrast with the findings of Rodriguez et al. [30], who suggested that *E. globulus*, *R. communis*, and *M. citrina* species are the main sources of resources for the manufacture of propolis.

As mentioned above, with the implementation of this methodology, it was possible to obtain other analysis resources, such as ethanolic and hexanic extracts, which, in the case of EEP, were implemented in different research protocols, such as the analysis of antimicrobial activity, which resulted in inhibitory activity against *S. aureus* but not against *E. coli*, which is in agreement with several works [25] reporting that the inhibitory action of propolis is affected by the formation of the usual cell wall in this type of bacteria, as well as the absence of certain components that could be responsible for the antibacterial activity. Thus, the absence or presence of the components is largely defined by the biotic and abiotic resources in the area or region where the apiary is located [2,36].

The results of both the palynological and physicochemical analyses of the same sample allowed for greater certainty in the comparison of results, in contrast with those obtained by Rodriguez et al. [30], which, unlike this research, showed that propolis from the same location presents inhibitory activity against Gram (+), Gram (−) bacteria as well as against yeasts such as *C. albicans*, whereas in our results, such activity was observed only with *S. aureus.* This is largely due to the vegetation source of resources for the manufacture of this product, as well as the climatic conditions, season, and time of the year of foraging and collection of propolis [2,41]. Moreover, this finding explains the absence of antifungal activity against *C. albicans* in the studied propolis [25,30,42].

In terms of quality, the phenolic load is a relevant factor since the antioxidant activity of these compounds contributes to the maintenance and recovery of human health [36,37,38]. The results obtained for phenols such as flavonoids, as well as IC_50_ in comparison with those obtained by Rodriguez et al. [30], are lower; however, they comply with the values requested in the Mexican standard [43], as well as in the Argentine and Brazilian statutes [26], which indicates that it is good-quality propolis. However, optical analysis revealed the presence of both fungal spores and yeasts, and their identification and quantification could reduce the quality of this propolis. In turn, the quality of the ethanolic extracts obtained allowed for HPLC-DAD, HPLC-MS, and CG-MS analysis, showing similar compounds between analyses that coincide with various investigations [44,45], coinciding in components such as pinocembrin and chrysin regular flavones in propolis temperate zones.

Other protocols that benefit from the implementation of this method include those of molecular genetics, since for implementing the necessary techniques for genetic determination, the integrity of the biological material, especially the internal content of the pollen grain, is necessary. Therefore, the method becomes a practical tool since it safeguards the biological integrity of the pollen, which allows the use of DNA extraction methodologies, which can be implemented in metagenomic studies [11,12,46].

## 5. Conclusions

In conclusion, this method represents a practical tool for achieving accurate and complete characterization of propolis. The application of our method allows us to obtain clean pollen under optimal conditions for pollen analysis, which can be used to classify propolis and determine its botanical origin. In addition, the use of this method can be useful for the analysis of propolis for other research purposes, such as the biomedical and physicochemical properties of propolis, because this technique provides materials such as extracts that are useful in other disciplines, such as pharmacognosy, phytochemistry, chemistry, and cosmetics.

## Figures and Tables

**Table 1:** Palynological spectrum obtained from the analysis of the sample processed by Soxhlet extraction.

Family	Genus	Species
Amaranthaceae	*Amaranthus*	*Amaranthus hybridus* L. (IZTA 3801)
Anacardiaceae	*Schinus*	*Schinus molle* L. (IZTA 3802)
Asteraceae	*Bidens*	*Bidens odorata* Cav. (IZTA 3806)
	*Taraxacum*	*Taraxacum campylodes* G.E. Haglund (IZTA 3811)
	*Titonia*	*Titonia tuubeaformis* (Jacq.) Cass. (IZTA 3812)
Brassicaceae	*Brassica*	*Brassica rapa* L. (IZTA 3814)
Convulvulaceae	*Ipomoea*	
Euphorbiaceae	*Ricinus*	*Ricinus communis* L. (FESC-6294)
Fabaceae	*Medicago*	*Medicago sativa* L. (IZTA 3817)
Moraceae	*Ficus*	
Myrtaceae	*Callistemon*	*Melaleuca citrina* (Curtis) Dum. Cours (IZTA 3820)
	*Eucalyptus*	*Eucalyptus globulus* Labill. (FESC-11044)
Olaceae	*Fraxinus*	*Fraxinus uhdei* (Wenz.) Lingelsh (IZTA 42812)
	*Ligustrum*	*Ligustrum japonicum* Thumb (IZTA 42812)
Pinaceae	*Pinus*	
Poaceae	*Zea*	*Zae mays* L.
Rosaceae	*Pronus*	

**Table 2:** The percentage of pollen types determined was obtained from the analysis of the sample processed by Soxhlet.

Pollen Type		%
*Brassica rapa* L.		40.00
*Melaleuca citrina* (Curtis) Dum. Cours		10.56
*Bidens odorata* Cav.		8.44
*Eucalyptus*		6.22
*Medicago sativa* L.		5.44
*Schinus molle* L.		4.44
*Titonia tuubeaformis* (Jacq.) Cass.		4.22
*Ricinus communis* L.		4.11
Complementary	*Pronus*	3.33
	*Fraxinus uhdei* (Wenz.) Lingelsh	2.56
	*Amaranthus hybridus* L.	2.00
	*Ligustrum japonicum* Thumb	2.00
	*Ipomoea*	1.89
	*Ficus*	1.67
	*Taraxacum campylodes* G.E. Haglund	1.44
	*Zae mays* L.	1.22
	*Pinus*	0.44

**Table 3:** Palynological spectrum by means of the component disaggregation method.

Family	Genus	Species
Altingiaceae	*Liquidambar*	
Amaranthaceae	*Amaranthus*	*Amaranthus hybridus* L. (IZTA 3801)
Anacardiaceae	*Schinus*	*Schinus molle* L. (IZTA 3802)
Asparagaceae	*Yucca*	
Asteraceae	*Bidens*	*Bidens aurea* (Aiton) Sherff (IZTA 3805)
		*Bidens odorata* Cav. (IZTA 3806)
		*Bidens pilosa* L. (IZTA 3807)
	*Helminthotheca*	*Helminthotheca echioides* (L) Holub (IZTA 3809)
	*Helianthus*	
	*Taraxacum*	*Taraxacum campylodes* G.E. Haglund (IZTA 3811)
Betulacaeae	*Alnus*	
Brassicaceae	*Brassica*	*Brassica rapa* L. (IZTA 3814)
	*Raphanus*	*Raphanus raphanistrum* L. (IZTA 3815)
	*Sisymbrium*	*Sisymbrium irio* L. (IZTA 3816)
Burseraceae	*Bursera*	
Casuarinaceae	*Casuarina*	*Casuarina equisetifolia* L.
Convulvulaceae	*Ipomoea*	
Cucurbitaceae	*Sicyus*	
Cupressaceae	*Cupressus*	
Euphorbiaceae	*Ricinus*	*Ricinus communis* L.(FESC-6294)
Fabaceae	*Acacia*	
	*Medicago*	*Medicago sativa* L. (IZTA 3817)
	*Trifolium*	*Trifolium amabile* Kunth (IZTA 3818)
Moraceae	*Ficus*	
Myrtaceae	*Callistemun*	*Melaleuca citrina* (Curtis) Dum. Cours (IZTA 3820)
	*Eucalyptus*	*Eucalyptus globulus* Labill (FESC-11044)
Olaceae	*Fraxinus*	*Fraxinus uhdei* (Wenz.) Lingelsh (IZTA 42812)
	*Ligustrum*	*Ligustrum japonicum* Thumb (IZTA 42812)
Pinaceae	*Pinus*	
Poaceae	*Zea*	*Zea mays* L.
Rosaceae	*Pronus*	
Salicaceae	*Populus*	
Solanaceae	*Nicotiana*	*Nicotiana glauca* Graham (IZTA 3831)
	*Solanum*	*Solanum rostratum* Dunal (IZTA 3833)

**Table 4:** Percentage of pollen types obtained through the method of component disaggregation.

Pollen Type		%
*Brassica rapa* L.		63.08
*Melaleuca citrina* (Curtis) Dum. Cours		5.00
*Eucalyptus globulus* Labill		4.83
*Fraxinus uhdei* (Wenz.) Lingelsh		4.00
*Trifolium amabile* Kunth		3.75
*Cupresus*		3.17
*Ricinus communis* L.		2.83
*Acacia*		2.00
*Alnus*		1.75
*C. equisetifolia* L.		1.75
*Schinus molle* L.		1.08
Complementary	*Ficus*	0.83
	*Pinus*	0.83
	*Helianthus*	0.75
	*Ipomoea*	0.75
	*Nicotiana*	0.67
	*Taraxacum*	0.67
	*Liquidambar*	0.33
	*Bidens*	0.33
	Amarantaceae	0.33
	*Pronus*	0.25
	*Yucca*	0.25
	*Sicyos*	0.25
	*Populus*	0.17
	*Zea maiz*	0.17
	*Ligustrum*	0.08
	*Bursera*	0.08
	Total complementary	6.75

**Table 5:** HPLC-DAD and HPLC-MS analysis of ethanolic extract of propolis from Cuautitlan.

Compound	Retention Time		λmax (nm)	Parention (m/z) [M-H]−	Relative Error (ppm)
	HPLC-DAD	HPLC-MS			
Naringin	2.494		220, 284		
Cantechol	2.494		216, 270		
Naringenin	4.096	20.49	224, 282	271.03	3.1
Kaempferol	7.406		220, 366		
Chrysin	12.284	28.08	268, 314, 348	253.03	−1.58
Luteolin		23.25		285.02	−10.74
Pinocembrin		26.55		255.04	3.95

**Table 6:** Compounds present in Cuautitlan propolis identified by gas chromatography–mass spectrometry.

Compound	RT (min)	%
Benzyl methyl ketone	7.92	1.12
m-Eugenol	11.93	3.97
Alpha bisabolol	16.91	1.06
Palmitic acid, methyl ester	19.97	7.75
Palmitic acid, ethyl ester	20.80	0.91
Linoleic acid, methyl ester	22.02	1.17
Oleic acid, methyl ester	22.09	9.33
Methyl stearate	22.36	1.94
Oleic acid, ethyl ester	22.84	0.94
Palmitamide	23.02	1.14
Methyl 18-methylnonadecanoate	24.56	2.29
Stearamide	25.24	0.97
Pinocembrin	26.51	30.29
Chrysin	28.39	15.77
6-O-Methylemodin, physcion	28.70	1.94
Galangin	28.83	5.67

## Data Availability

Data are contained within the article.

## Supplementary Materials

The following supporting information can be downloaded at: https://www.mdpi.com/article/10.3390/antiox13111412/s1
