# Supplementary material for: Determination of the Botanical Origin and Physicochemical Properties of a Propolis Sample Through an Integrated Methodology
Source: Antioxidants (Basel). 2024 Nov 18;13(11):1412. doi: 10.3390/antiox13111412 (PMC11591193; doi:10.3390/antiox13111412)

HPLC-TOF-MS

Mass spectrum of the compounds detected.

Unidentified compound

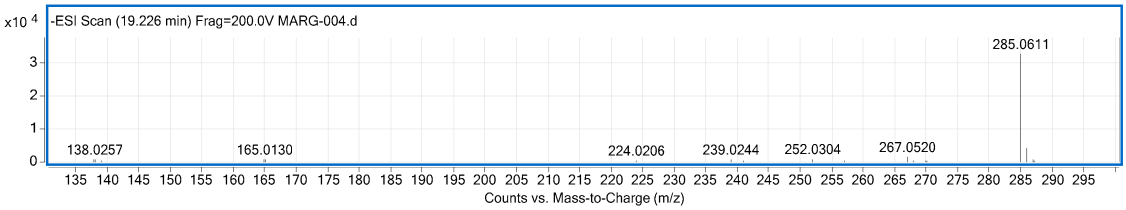

Naringin 271.0384 PPM 3.1

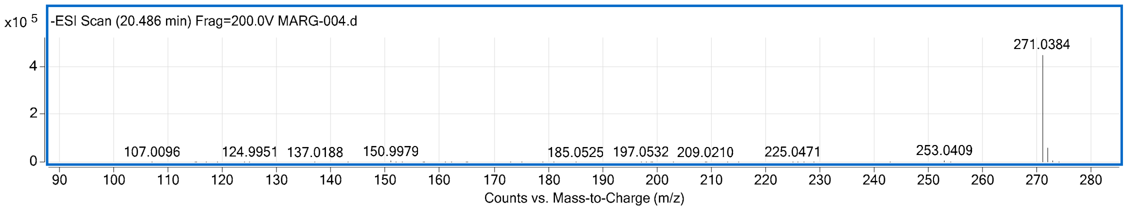

Luteolin 285.0283 PPM -10.74

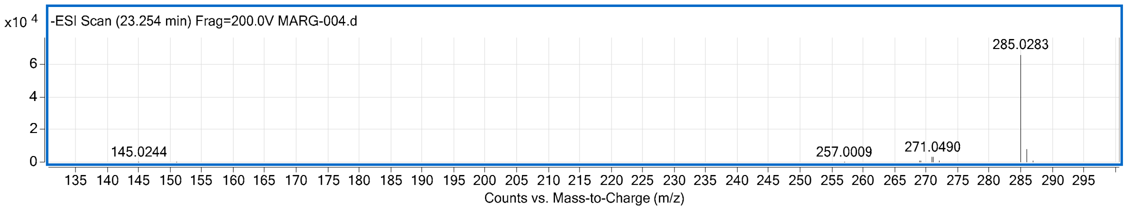

Unidentified compound

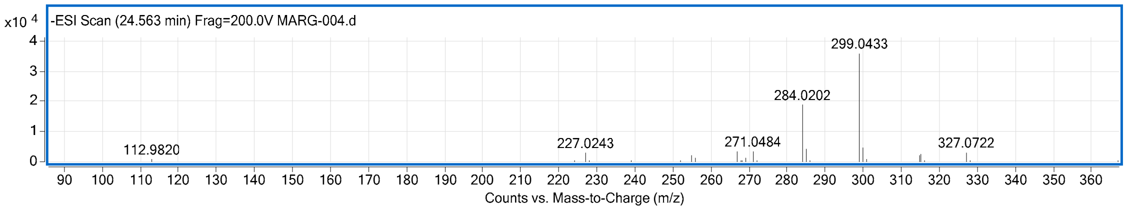

Unidentified compound

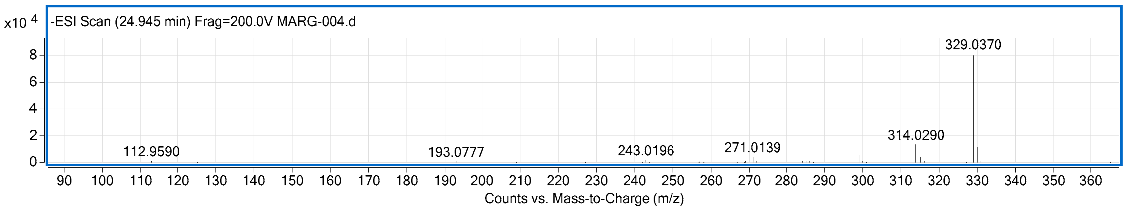

Pinocembrin 255.0406 PPM 3.95

Unidentified compound

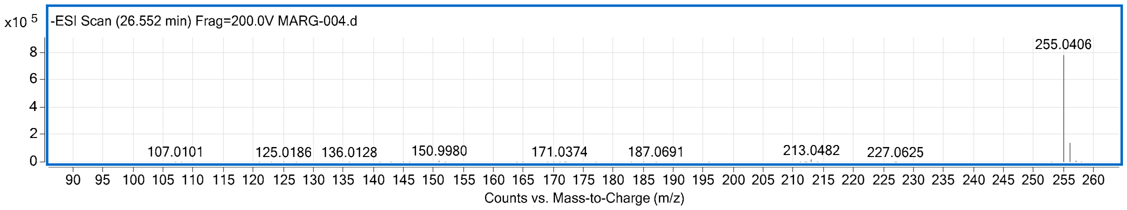

Chrysin 253.0301 PPM -1.58

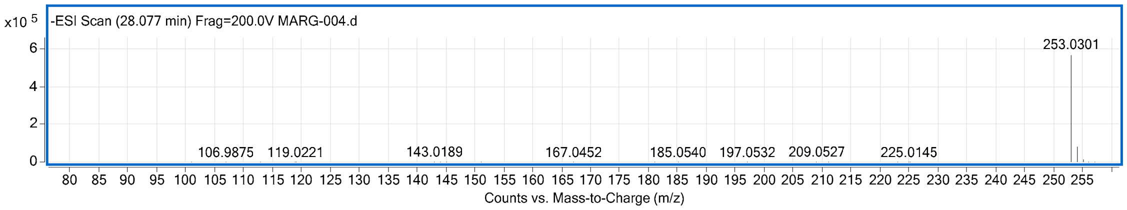

Unidentified compound

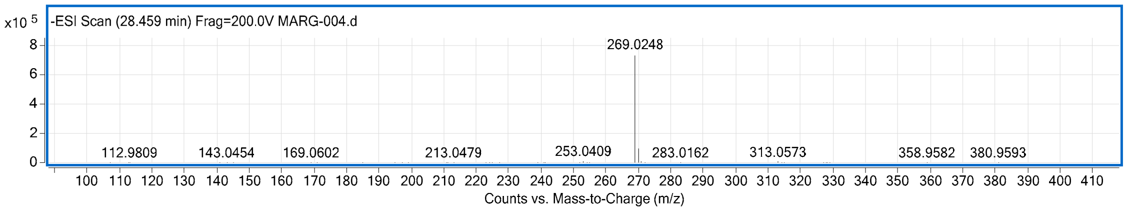

Unidentified compound

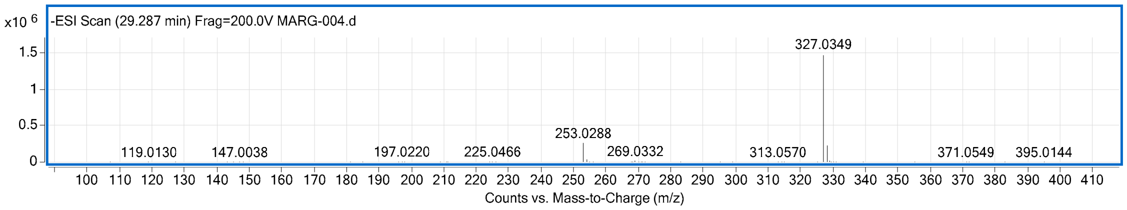

Unidentified compound

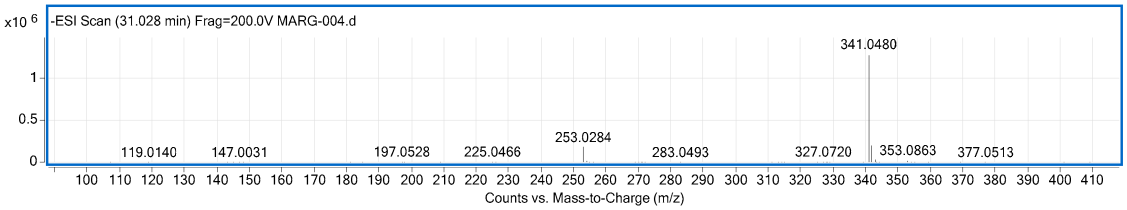

Unidentified compound

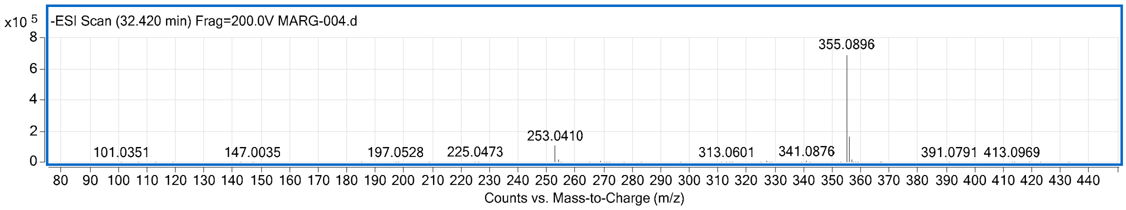

Unidentified compound

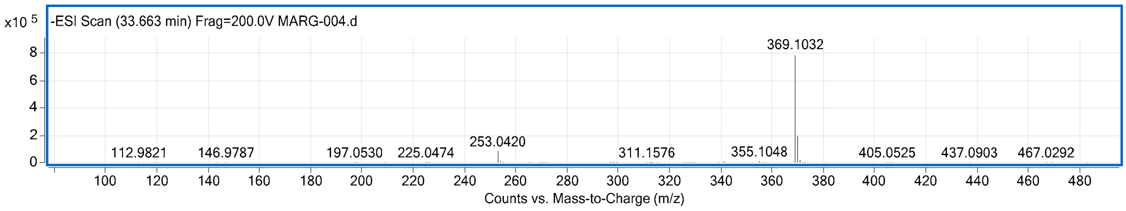

Unidentified compound

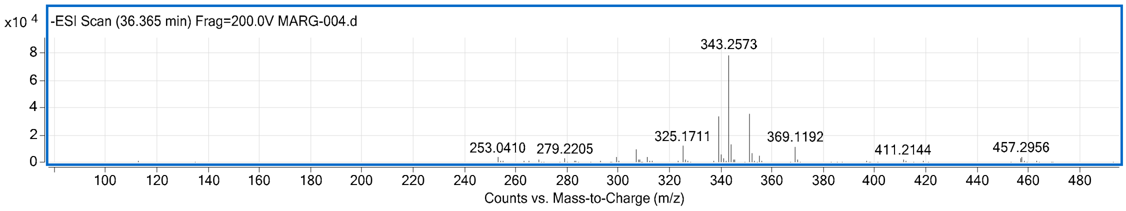

Unidentified compound

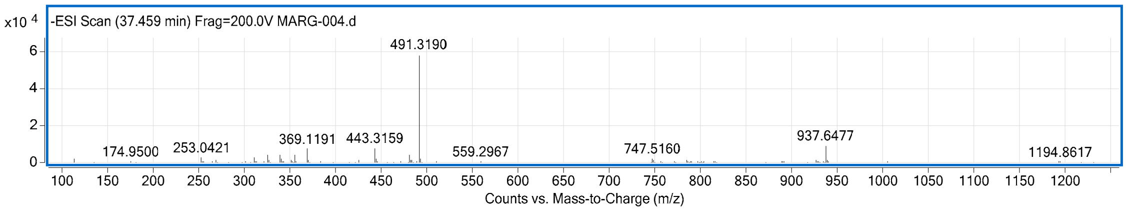

Unidentified compound

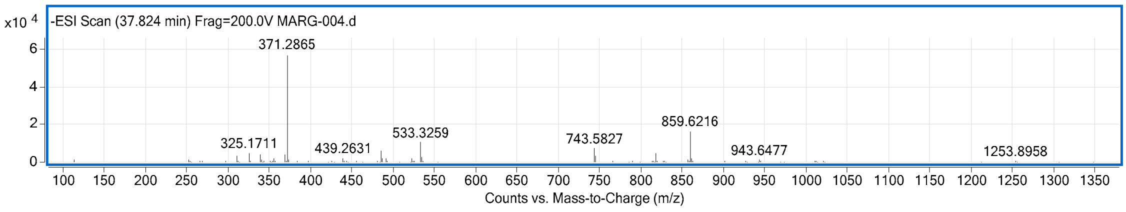

Supplement: Supplementary file 1 [file antioxidants-13-01412-s001.zip › antioxidants-3237865-supplementary.pdf]
